# Hydrocarbon‐degrading bacteria in deep‐water subarctic sediments (Faroe‐Shetland Channel)

**DOI:** 10.1111/jam.14030

**Published:** 2018-07-24

**Authors:** E. Gontikaki, L.D. Potts, J.A. Anderson, U. Witte

**Affiliations:** ^1^ School of Biological Sciences University of Aberdeen Aberdeen UK; ^2^ Surface Chemistry and Catalysis Group, Materials and Chemical Engineering, School of Engineering University of Aberdeen Aberdeen UK

**Keywords:** clone libraries, Faroe‐Shetland Channel, hydrocarbon degradation, isolates, marine bacteria, oil spill, *Oleispira*, sediment

## Abstract

**Aims:**

The aim of this study was the baseline description of oil‐degrading sediment bacteria along a depth transect in the Faroe‐Shetland Channel (FSC) and the identification of biomarker taxa for the detection of oil contamination in FSC sediments.

**Methods and Results:**

Oil‐degrading sediment bacteria from 135, 500 and 1000 m were enriched in cultures with crude oil as the sole carbon source (at 12, 5 and 0°C respectively). The enriched communities were studied using culture‐dependent and culture‐independent (clone libraries) techniques. Isolated bacterial strains were tested for hydrocarbon degradation capability. Bacterial isolates included well‐known oil‐degrading taxa and several that are reported in that capacity for the first time (*Sulfitobacter*,* Ahrensia, Belliella, Chryseobacterium*). The orders Oceanospirillales and Alteromonadales dominated clone libraries in all stations but significant differences occurred at genus level particularly between the shallow and the deep, cold‐water stations. *Alcanivorax* constituted 64% of clones at FSC135 but was absent at deeper stations. *Pseudoalteromonas* and *Oleispira* dominated the bacterial community at 500 and 1000 m.

**Conclusions:**

The genus *Oleispira* emerged as a major player in the early stages of crude oil degradation in deep‐sea sediments of the FSC particularly at subzero temperatures. This finding is offering a direction for future research into biomonitoring tools for the detection of low levels of crude oil contamination in the deep FSC, and possibly high latitude cold waters in general.

**Significance and Impact of the Study:**

Oil and gas exploration in the FSC occurs at depths >1000 m but baseline environmental data necessary for the assessment of ecosystem recovery to prespill conditions in the event of an oil spill are lacking. This study will contribute to our ability to assess the impact of oil release in the FSC and guide the direction of bioremediation strategies tailored to the area.

## Introduction

As much as 12% of oil is currently extracted from the deep sea (at depths >200 m), compared to 2% in 2001 (Larkin *et al*. 2015). Ultra‐deep drilling for oil and gas at depths >1000 m is still in early stages but exploration and exploitation of reserves at these depths is likely to increase in coming years as political and economic pressure mounts to access deep‐water fossil reserves (Scoma *et al*. 2016b). Recent empirical studies show that the probability of accidental oil release is positively correlated with drilling depth suggesting increased risks of deep‐sea oil spills in the future (Muehlenbachs *et al*. 2013). Nevertheless, the ability of the industry to contain oil spills in deep waters has not advanced in pace with drilling technologies while no broadly supported management strategy is in place to limit environmental impacts attributable to the deep‐sea oil and gas industry (Jernelöv 2010; Cordes *et al*. 2016).

Contamination of deep‐sea environments with petroleum following accidental spills represents an emerging issue, which received worldwide attention after the 2010 Deepwater Horizon (DWH) accident in the Gulf of Mexico (Scoma *et al*. 2016b). In that case, up to 50% of the discharged oil and essentially all of the natural gas was sequestered in a deep‐water hydrocarbon plume at 1000–1300 m depth (Joye 2015; Passow and Hetland 2016) while 2–15% of spilt oil was deposited on the Gulf seafloor mainly via the sedimentation of oil‐associated marine snow from surface waters (Passow *et al*. 2012; Chanton *et al*. 2014; Valentine *et al*. 2014; Ziervogel *et al*. 2016). Oil deposition on the deep seafloor represented a previously unrecognized fate of oil that should be factored into future oil spill budgets (Joye *et al*. 2014; Joye 2015; Daly *et al*. 2016). The ecological consequences of oil deposition on deep‐sea sediments are numerous; the resulting chemically reducing conditions that follow deposition have adverse effects on the biodiversity of sediment biota and cause cascading biogeochemical changes in affected sediments, which consequently alter the function and services provided by the ecosystem (Montagna *et al*. 2013; Brooks *et al*. 2015; Romero *et al*. 2015; Schwing *et al*. 2015). Of particular concern is the persistence of certain hydrocarbons in deep‐sea sediments for decades and the risk of prolonged ecotoxicological effects as these compounds gradually enter the food web (Yan *et al*. 2016).

Deep‐water microbial communities played a pivotal role in the DWH oil spill remediation; a succession of blooming hydrocarbon‐degrading bacteria including DWH Oceanospirillales, Colwellia and Cycloclasticus, removed an estimated 50% of the oil trapped in the deep plume (Joye 2015). Similarly, remarkable shifts in bacterial communities were further observed in deep‐sea sediments where *Cycloclasticus* represented the most persistent microbial marker of seafloor hydrocarbon deposition (Yang *et al*. 2016a). Sulphate‐reducing, anaerobic bacteria, such as Desulfobacteraceae and Desulfobulbaceae*,* also became enriched in response to the occurrence of anaerobic microniches in Gulf sediments (Yang *et al*. 2016a). While, the key role of biodegradation in the removal of oil from the marine environment is widely accepted, the natural potential of hydrocarbon‐degrading micro‐organisms for biotechnological applications is currently not realized due to severe gaps in our knowledge of the biology of naturally occurring marine oil‐degrading bacteria and their populations (Kube *et al*. 2013). The detection and identification of key micro‐organisms that respond to hydrocarbon inputs is the first step towards a site‐specific assessment of the bioremediation potential and appropriate response strategies in the event of anthropogenic hydrocarbon discharges (Joye *et al*. 2016). The aim of this study was to provide baseline information on the identity of key hydrocarbon‐degrading bacteria in sediments of the Faroe‐Shetland Channel (FSC), an active area of oil exploration and planned production at depths of 1100 m on the continental shelf of the United Kingdom (UKCS) (Austin *et al*.^2014^). To capture as much diversity as possible, enrichment of hydrocarbon degraders with crude oil was followed by identification of key bacteria using both culture‐dependent and independent techniques. The capability of bacterial isolates to degrade hydrocarbons was also assessed. The suitability of certain hydrocarbon‐degrading bacteria as biomarkers of oil contamination in cold, deep‐water sediments is discussed. The information provided in this study will improve the ability of the scientific community to assess the impact of anthropogenic oil release in the FSC and guide the direction of bioremediation strategies tailored to the area.

## Materials and methods

### Study site

The FSC is a deep‐water channel separating the Faroe Plateau from the Scottish shelf and constitutes a critical link between the North Atlantic and the Nordic Seas. The FSC is characterized by a particularly complex hydrography with northward‐flowing warm North Atlantic waters overlying cold‐water masses of Arctic origin flowing into the Rockall Trough and the wider deep North Atlantic Ocean. As a result, water temperature varies dramatically within only a few hundred metres, from 9–12°C in the top 200 m to subzero temperatures below 600 m (Berx 2012). Three sampling sites along a depth gradient in the FSC were chosen to reflect three major zones that are influenced by different water masses (Table 1). The water above ~400 m is made up of North Atlantic Water which flows as a slope current along the Shetland Shelf at temperatures of 10–12°C (station FSC135). Station FSC500 falls within a transition zone (typically around 400–800 m depth and temperature ranging between 2–6°C) where Modified East Icelandic Water and Norwegian Sea Arctic Intermediate Water can be found. FSC1000 is influenced by the deepest water mass (Norwegian Sea Deep Water) which makes up most of the southward overflow, directed through the Faroe Bank Channel and across the Wyville Thomson Ridge (Berx 2012).

**Table 1:** List of stations and coordinates

Station id.	Depth (m)	Lat (DD)	Long (DD)
FSC135	135	59·888	−3·348
FSC500	538	61·133	−2·173
FSC1000	994	61·233	−2·664

### Sediment collection

Sediments from 135 m in the FSC (station FSC135, Table 1) were kindly collected by Premier Oil Ltd using a ROV. The sediments were kept chilled until the initiation of enrichment cultures two weeks following collection. Sediments from 538 m and 994 m in the FSC (stations FSC500 and FSC1000 respectively) were collected using a day grab onboard the *FRV* Scotia (cruise number Sc201405) between 24 April 2014 and 8 May 2014. Sampling with a day grab was the best solution possible given the coarse sediments at shallow and mid depths in the FSC, and the limited ship time on FRV Scotia which prevented sampling with a multicorer at FSC1000. Sediment from five grabs collected at each station was pooled and stored at 1°C under approximately 10 cm of bottom water from the respective station. The overlying water was constantly aerated until enrichment cultures were initiated. The background composition of the bacterial community in sediments from FSC500 and FSC1000 is published elsewhere (Ferguson *et al*. 2017).

### Media and substrates

Enrichment of HDB communities was performed using the ONR7 medium (Dyksterhouse *et al*. 1995). Stock solutions of the PAHs naphthalene, phenanthrene, pyrene, fluorene and anthracene were prepared in acetone at a final concentration of 10 g l^−1^. Stock solutions were sterilized by filtration through a 0·2 *μ*m membrane filter. PAHs (≥99·0%) were purchased from Sigma‐Aldrich, Inc. (St. Louis, MO, USA). Crude oil (autoclave sterilized) from the Schiehallion oilfield was also used as a substrate. A mixture of 20 hydrocarbons which included both aliphatic and aromatic compounds (‘model oil’, filter‐sterilized) was used in the assay for the determination of the hydrocarbon degradation capability of isolates. The exact composition of the hydrocarbon mix is described elsewhere (Ferguson *et al*. 2017).

### Enrichment cultures

PAHs, either in isolation or as mixture, and crude oil were used as substrates in enrichment cultures and served as the sole carbon and energy source for growth. The following treatments were prepared: naphthalene (NAP), phenanthrene (PHE), pyrene (PYR), PAH mix (naphthalene, phenanthrene, pyrene, anthracene, fluorene) and crude oil (CRU). The PAHs were added to empty 50 ml Erlenmeyer flasks and the solvent was allowed to evaporate (Table S1). An amount of 30 ml of ONR7 medium was subsequently added followed by 1 g of sediment which served as the inoculum. Crude oil was added after the ONR7 medium at 1% (v/v) concentration. Controls without carbon source addition were also prepared for each treatment. Two successive enrichments were prepared with 1% (v/v) culture used as the inoculum for each subculture at 3‐week intervals. The cultures were incubated aerobically at 12, 5 and 0°C for FSC135, FSC500, FSC1000 respectively corresponding to ambient temperatures for each station. Aliquots of the enriched HDB communities after the end of the incubation period were stored at −80°C in final concentration of 20% glycerol.

### Strain isolation and identification

To obtain hydrocarbon‐degrading isolates, the final enrichments were diluted in ONR7 medium, plated onto ONR7 agar plates and sprayed with the same PAH used in the enrichment. Naphthalene was supplied as vapour after spraying the PAH on the petri dish lid. Crude oil (1 : 1 v/v in diethyl ether) was spread on the agar prior to the inoculant. Diluted cultures were also spread on Difco™ Marine Agar 2216 and ONR7 agar without substrate addition as controls. Individual colonies from all plates were picked with sterile pipette tips and were streaked on Marine Agar 2216 for purification. Genomic DNA from isolates was obtained using the DNeasy UltraClean Microbial DNA extraction kit (Qiagen, Hilden, Germany). *16S* rRNA genes from isolates were PCR amplified from genomic DNA with primers 27F (Lane 1991) and 1492R (Vergin *et al*. 1998) (10 *μ*mol l^−1^) using Red Taq DNA Polymerase Master Mix (2·0 mmol l^−1^ MgCl_2_; VWR, Radnor, PA, USA) and 1 *μ*l of template DNA. Amplification was performed on a Techne thermal cycler as follows: initial denaturation at 95°C for 5 min followed by 25 cycles of 1 min at 95°C, 1 min annealing at 55°C, 1 : 40 min primer extension at 72°C, and a final extension at 72°C for 10 min (Leigh *et al*. 2010).

### Hydrocarbon degradation capability of pure bacterial strains

Each isolate was tested for their ability to degrade hydrocarbons (described as positive or negative) using a modified version of the Wrenn and Venosa (1996) method for MPN enumeration of hydrocarbon‐degrading bacteria. Tests were performed in 96‐well plates filled with ONR7 medium (193 *μ*l) and ‘model oil’ as substrate (2% v/v). An amount of 1 ml of enrichment culture was centrifuged and washed three times in 1x PBS to remove culture broth. The pellet was resuspended in 1 ml 1x PBS and 5 *μ*l was added to each well. Two replicate wells per strain were prepared and 50 *μ*l of iodonitrotetrazolium chloride (INT; 3 g l^−1^) was added to each well after two weeks of incubation at 20°C. In positive wells, INT is reduced to an insoluble formazan that deposits intracellularly as a red precipitate. Positive wells were scored after overnight incubation with INT at room temperature (20°C).

### Culture‐independent microbial community analysis

Clone libraries of the *16S* rRNA gene were constructed from a set of revived enrichment cultures. The glycerol‐preserved HDB communities enriched with crude oil from all stations were revived in Difco Marine Broth 2216. The effectiveness of glycerol cryopreservation on mixed communities is currently unknown (Prakash *et al*. 2013). Nevertheless, a recent study demonstrated consistent preservation of dominant micro‐organisms in complex communities using this preservation method (Yu *et al*. 2015) and hydrocarbon‐degrading communities revived from glycerol stocks have been used previously in incubation experiments (Campo *et al*. 2013; Techtmann *et al*. 2017). Analysis of the initial composition of revived communities from FSC135 and FSC1000 after 30 h of incubation (20°C) is given in Potts *et al*. (2018). Briefly, the revived community at the shallow station was dominated by *Pseudoalteromonas* (47%), followed by *Halomonas* (19%), *Albirhodobacter* (12%), *Pseudomonas* (6%), *Colwellia* (6%), *Brumimicrobium* (4%) and *Alcanivorax* (3%). The most abundant taxa of the revived community at FSC1000 were *Pseudoalteromonas* (40%), *Pseudomonas* (27%), *Bizionia* (22%), unclassified Rhodobacteraceae (5%), *Thalassospira* (2%) and *Marinomonas* (1%). *Alcanivorax* was present at very low abundance at the FSC1000 revived community (0·01%). *Oleispira* abundance was at 0·002% in both revived communities. The revived cultures were used to inoculate two replicate flasks of ONR7 medium and crude oil for each station as previously described. The flasks were incubated on a shaking table for 3 weeks at the *in situ* temperature for each station. At the end of the incubation period, 10 ml of culture was concentrated to 400 *μ*l by centrifugation (1100 ***g*** for 10 min) and resuspension of the pellet in 1x PBS. Total bacterial DNA was extracted from the concentrated biomass using the FastDNA™ SPIN Kit for Soil and the FastPrep^®^ Instrument (MP Biomedicals, Santa Ana, CA). DNA from replicate flasks was combined and stored at −20°C until further analysis. The *16S* rRNA gene was amplified using the 27F/1492R primer pair and a PCR program as above. Agarose gel electrophoresis (1·2% agarose, 100V, 30–45 min) verified the success of PCR amplification based on the predicted amplicon size. The gels were stained with GelRed™ (VWR) and visualized on a UV transilluminator (U:Genius^3^; Syngene, India). PCR products were purified using the Thermo Scientific GeneJET PCR Purification Kit (Thermo Fisher Scientific Inc., Waltham, MA, USA) according to manufacturer's instructions. Purified PCR products were ligated into the pGEM^®^‐T Easy cloning vector and transformed into RapidTrans™ chemically competent *Escherichia coli* cells as recommended by the manufacturer (pGEM‐T Easy Vector Systems; Promega, Madison, WI, USA). Successful cloning of inserts into the pGEM‐T Easy cloning vector was screened on MacConkey agar plates prepared with ampicillin at a final concentration of 100 mg l^−1^. A volume of 50 and 100 *μ*l of each transformation culture was spread on duplicate MacConkey/ampicillin agar plates which were incubated overnight at 37°C. Following incubation, colonies on the plates were either red or creamy white, with the latter indicating clones containing an insert. Each clone was grown in LB broth/ampicillin (100 mg l^−1^) medium in a 96‐well plate. Following overnight incubation at 37°C, 1 *μ*l of each liquid culture served as template for PCR amplification of the cloned *16S* rRNA genes in 50 *μ*l reactions using the vector primers M13f and M13r. PCR programme was as follows: initial denaturation at 94°C for 5 min followed by 25 cycles of 30 s at 94°C, 1 min annealing at 55°C, 2 min primer extension at 72°C, and a final extension at 72°C for 5 min. PCR products were electrophoresed on a 1·2% agarose gel and clones with abnormally sized inserts were removed from further analysis. PCR products were screened with restriction fragment length polymorphism (RFLP) to identify replicate clones in the library following the protocol of Vergin *et al*. (2001). An amount of 10 *μ*l of PCR product was digested with *Bsu*RI (*Hae*III) (Thermo Scientific) according to manufacturer's instructions. The digestion products were visualized on a 2% agarose gel and the clones were distributed into RFLP groups. The procedure was repeated with the enzyme *Mbo*I (Thermo Scientific). Clones belonging to the same RFLP group based on *Bsu*RI were compared in adjacent lanes of a second 2% agarose to verify identical clones. Any clones that produced different RFLP patterns with *Mbo*RI were placed in a new RFLP group. Selected clones were sequenced using the 27F primer. Sequencing of several clones corresponding to dominant RFLP types revealed that these RFLP groups were internally homogeneous (data not shown). All clones belonging to RFLP groups with less than five representatives were sent for sequencing.

### Crude oil degradation by enriched consortia

The degradation of crude oil by the revived consortia after three weeks of incubation was measured by gravimetric analysis. Residual hydrocarbons were extracted from flasks using 10 ml dichloromethane (DCM) in six sequential extractions. The procedure was as follows: 10 ml DCM was added to the flask and agitated manually for 30 s. Solvent/media mixture was then decanted into a separating funnel and shaken vigorously then left to separate for 10 min. Following separation, the organic phase was drained into a preweighed beaker. This was repeated five further times, each time rinsing the incubation flask with solvent before adding it to the separating funnel. After extraction, DCM was left to evaporate from the beaker, which was reweighed until consistent weight. Residual oil was calculated as the difference in the weight of the flask before and after extraction. The amount of degraded crude oil was calculated by extracting the weight of residual oil from the total amount of oil added in the flask at the beginning of the incubation.

### Bioinformatics

Consensus sequences from matching forward and reverse traces were computed using the SeqTrace software (isolates only) (Stucky 2012). Quality‐trimming and vector removal were performed using the RDP Sanger pipeline (Cole *et al*. 2014). Sequences were compared to entries in various sequence databases using the SILVA Incremental Aligner (sina ver. 1.2.11) (Pruesse *et al*. 2007). The evolutionary history of bacterial clones from the FSC and nearest neighbour sequences was inferred by using the Maximum Likelihood method based on the Kimura 2‐parameter model (Kimura 1980). The percentage of trees in which the associated taxa clustered together in the bootstrap test (100 replicates) is shown next to the branches. The tree is drawn to scale, with branch lengths measured in the number of substitutions per site. Evolutionary analyses were conducted in mega7 (Kumar *et al*. 2016).

### Nucleotide sequence accession numbers

The nucleotide sequences determined in this study have been deposited in the GenBank database under accession numbers MF977460 to MF977503 for isolates and MF978430 to MF978509 for clones sequences.

## Results

### Isolates

All isolated strains obtained from enrichment cultures were subjected to sequence analysis of the *16S* rRNA gene. In FSC135, 13 isolates were analysed and belonged to eight genera of the Gamma‐ (38·5%) and Alpha‐ (23·1%) Proteobacteria, Bacteroidetes (23·1%) and Actinobacteria (15·4%) (Table 2). Strains closely related to *Halomonas* sp. were dominant among isolated strains. Several isolates obtained from FSC135 were unique among stations; strains closely related to *Ahrensia* sp. (Rhodobacterales, Alphaproteobacteria), the genera *Belliella*,* Aequorivita* and *Brumimicrobium* (Bacteroidetes), and the family Kineosporiaceae (Actinobacteria) were not found at deeper stations. A striking difference of FSC135 compared to FSC500 and FSC1000 was the absence of the order Alteromonadales and particularly *Pseudoalteromonas,* which was common in the deep stations. *Shewanella*‐ and *Glaciecola*‐type strains were isolated from both deep stations while *Colwellia* was found only at FSC1000. *Rhodococcus* sp. (Actinobacteria) was the only common genus between all three stations and showed strong potential for hydrocarbon degradation (Table 2).

**Table 2:** Bacterial isolates from FSC135, FSC500 and FSC1000 and HC degradation capability. Origin culture denotes the treatment (NAP, PHE, PYR, PAH mix, CRUDE) each strain was isolated from. Testing for hydrocarbon degradation was performed using model oil as substrate, which contains a mixture of aliphatic and aromatic hydrocarbons

Isolate id. (accession number)	Origin culture	Query length	Closest relative in SILVA database (accession number)	Origin of closest neighbour	Similarity (%)	Hydrocarbon degradation
135‐A (MF977460)	NAP	1358	*Belliela aquatica* TS‐T86 (KC762321)	Culture of the alga *Phaeodactylum tricornutum*, China	92%	+
135‐B (MF977461)	CRUDE	1384	*Aequorivita viscosa* 81‐b (HM485318)	Intertidal zone, East China Sea	97%	−
135‐C (MF977462)	PYR	1387	Uncultured *Halomonas* sp. clone C146300069 (JX530673)	Southern ocean iron fertilization experiment (LOHAFEX)	99%	+
135‐D (MF977463)	PYR	1394	*Halomonas* sp. B01 (KJ778559)	Salt pond sediment of a seawater baysalt field	99%	+
135‐E (MF977464)	PYR	943	Uncultured *Halomonas* sp. clone C146300069 (JX530673)		99%	+
135‐F (MF977465)	PYR	948	Uncultured *Halomonas* sp. clone C146300069 (JX530673)		99%	+
135‐G (MF977466)	CRUDE	1400	*Pseudomonas pelagia* CL‐AP6 (AROI01000066)	Culture of *Pyramimonas gelidicola* from the Antarctic	98%	++
135‐H (MF977467)	NAP	1341	*Ahrensia kielensis* DSM 5890 (ARFW01000003)		98%	++
135‐I (MF977468)	NAP	1347	*Ahrensia kielensis* NBRC 15762 (AB680960)		98%	−
135‐J (MF977469)	NAP	1299	*Sulfitobacter undariae* strain W‐BA2 (KM275624)	Brown algae reservoir, South Korea	99%	+
135‐K (MF977470)	NAP	1385	*Brumimicrobium* sp. P99 (EU195945)	Hydrocarbon polluted sediments, Spain	96%	−
135‐L (MF977471)	CRUDE	1376	*Kineosporiaceae* bacterium G3A1 (KF994923)		94%	+
135‐M (MF977472)	CRUDE	1346	*Rhodococcus fascians* strain Gert_13:10 (KR088385)	Groundwater	100	++
500‐A (MF977473)	PHE	1366	*Chryseobacterium* sp. LW‐NC3 (KJ958494)	Wastewater	98%	++
500‐B (MF977474)	PHE	1395	*Pseudomonas* sp. gap‐f‐76 (DQ530477)	Crude oil‐contaminated Antarctic sea‐ice	100%	+
500‐C (MF977475)	PHE	1392	*Pseudoalteromonas haloplanktis* TB25 ctg310 (AUTI01000310)	Antarctic sponge	100%	+
500‐D (MF977476)	CRUDE	1391	*Pseudoalteromonas haloplanktis* TAE80 (AUTM01000360)	Antarctic water column	100%	+
500‐E (MF977477)	CRUDE	1391	*Pseudoalteromonas haloplanktis* TAE80 (AUTM01000360)	Antarctic water column	100%	−
500‐F (MF977478)	PYR	1393	*Pseudoalteromonas elyakovii* (AF082562)		100%	+
500‐G (MF977479)	PHE	1395	*Pseudoalteromonas haloplanktis* TAE79 (AUTL01000217)	Antarctic water column	100%	+
500‐H (MF977480)	PAH	1397	*Shewanella* sp. 135Z‐7 (JX310137)	Southern Ocean	100%	−
500‐I (MF977481)	PYR	1397	*Shewanella vesiculosa* (AM980877)	Antarctic sediment	100%	−
500‐J (MF977482)	PHE	1384	*Glaciecola polaris* LMG 21857 (AJ29382)	Polar seas	100%	−
500‐K (MF977483)	CRUDE	1321	*Sulfitobacter* sp. PIC‐72 (AJ534241)	North Sea	100%	+
500‐L (MF977484)	CRUDE	1326	*Sulfitobacter* sp. BSw21498 (GQ358930)	Subarctic glacial fjord, Kongsfjorden	100%	+
500‐M (MF977485)	PYR	1365	*Rhodococcus* sp. R7704 (AJ295711)	Polar seas	100%	++
1000‐A (MF977486)	PAH mix	1377	*Shewanella* sp. JL‐56 (AY745827)	North pacific	100%	−
1000‐B (MF977487)	PAH mix	1398	*Shewanella* sp. R7216 (AJ295714)	Polar seas	100%	−
1000‐C (MF977488)	CRUDE	1405	*Shewanella* sp. 135Z‐7 (JX310137)	Southern Ocean	100%	
1000‐D (MF977489)	PAH mix	1393	*Pseudoalteromonas haloplanktis* TB13 (AUTJ01000235)	Antarctic sponge	100%	−
1000‐E (MF977490)	PAH mix	1363	*Pseudoalteromonas* sp. An83 (LN881340)	Surface microbiota of the brown alga *Ascophyllum nodosum*	100%	+
1000‐F (MF977491	PAH mix	1361	*Pseudoalteromonas* sp. S8‐8 ctg46 (AUTR01000046)	Antarctic sediment	100%	+
1000‐G (MF977492)	CRUDE	1366	*Pseudoalteromonas flavipulchra* (JTDZ01000010)	Seawater, Nice, France	100%	+
1000‐H (MF977493)	CRUDE	1376	*Pseudoalteromonas* sp. BIc20004 (FJ748504)	Ny‐Alesund ice core	100%	−
1000‐I (MF977494)	CRUDE	1376	*Pseudoalteromonas* sp. An93 (LN881244)	Surface microbiota of the brown alga *Ascophyllum nodosum*	100%	−
1000‐K (MF977495)	CRUDE	1384	*Pseudoalteromonas* sp. B36 (AB607158)	North Sea seawater	100%	+
1000‐L (MF977496)	PYR	1382	*Pseudoalteromonas* sp. BSw20083 (EU365608)	Arctic seawater	100%	−
1000‐M (MF977497)	PHE	1392	*Pseudoalteromonas elyakovii* (AF082562)		100%	−
1000‐N (MF977498)	CRUDE	1381	*Colwellia* sp. SS12.12 (KC160905)	Antarctic sediment	99%	−
1000‐O (MF977499)	PYR	1391	*Glaciecola polaris* LMG 21857 (AJ293820)	Polar Seas	100%	−
1000‐P (MF977500)	PYR	1380	*Marinomonas* sp. BSw10506 (EF437161)	Antarctic seawater	98%	−
1000‐Q (MF977501)	CRUDE	1377	*Bizionia* sp. KJF12‐2 (JQ800199)	Subarctic glacial fjord, Kongsfjorden	98%	−
1000‐R (MF977502)	CRUDE	1344	*Bizionia* sp. KJF12‐2 (JQ800199)	Subarctic glacial fjord, Kongsfjorden	98%	−
1000‐S (MF977503)	CRUDE	1370	*Rhodococcus* sp. NS8 (KM361868)	Nepheline sands from tailing dumps, Russia	100%	++

### Clones

**Figure 1:**
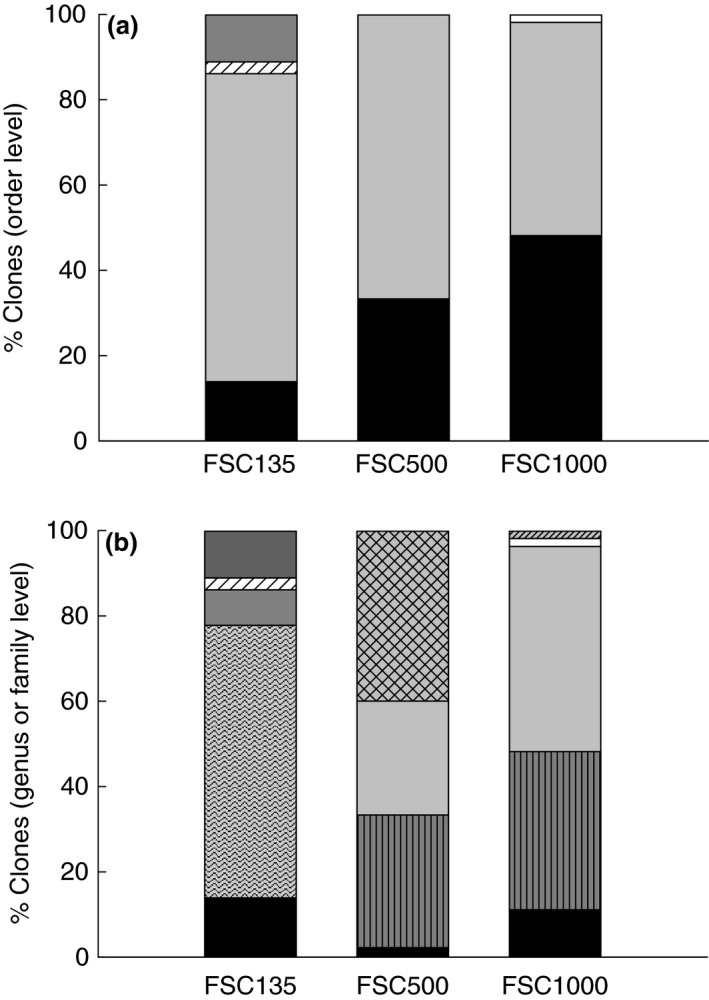
Relative abundance of clones at Order (a: 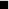 Alteromonadales; 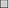 Oceanospirillales; 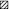 Pseudomonadales; 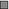 Rhodobacterales; 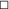 Flavobacteriales) and Genus (or Family in the case of Rhodobacteraceae) (b: 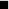 Colwellia; 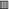 Pseudoalteromonas; 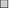 Oleispira; 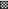 Marinomonas; 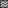 Alcanivorax; 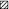 Halomonas; 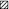 Profundimonas; 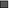 Pseudomonas; 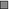 Rhodobacteraceae; 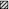 Bizionia) level in enrichment cultures after 3 weeks of incubation at ambient, for each station, temperature (12, 5 and 0°C for FSC135, FSC500 and FSC1000, respectively).

**Figure 2:**
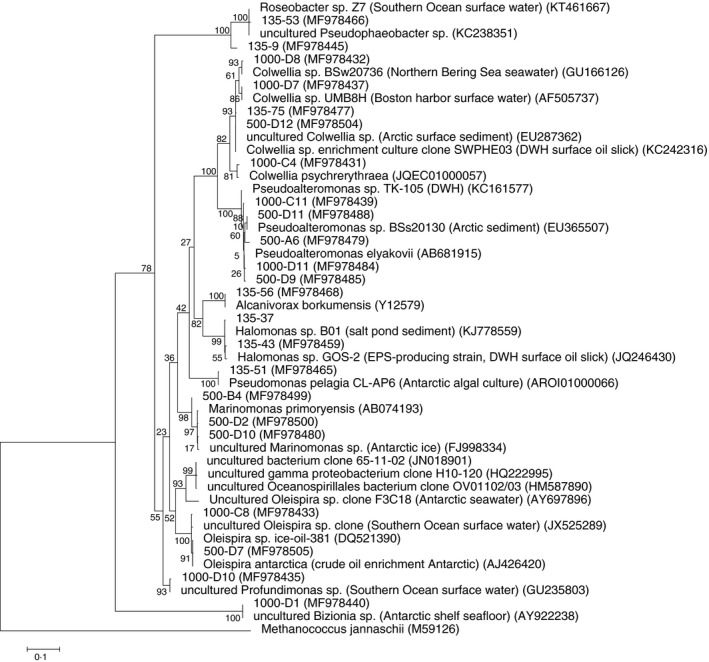
Phylogenetic tree illustrating the evolutionary relationship of bacterial clones from FSC135, FSC500 and FSC1000 stations in the Faroe‐Shetland Channel and nearest neighbour sequences (accession numbers in parenthesis). The evolutionary history was inferred using the Maximum Likelihood method. The percentage of replicate trees in which the associated taxa clustered together in the bootstrap test (100 replicates) is shown next to the branches. *Methanococcus jannaschii* (M59126) was used as the outgroup. Sequences marked with an asterisk are representative of a group of identical clones.

Clone libraries of PCR‐amplified *16S* rRNA genes from enrichment cultures with crude oil as the only carbon and energy source were constructed for all three stations. Insert PCR products were screened with RFLP to identify replicate clones in the libraries. The resulting RFLP patterns were used to group identical clones. In total, 70, 45 and 54 clones from FSC135, FSC500 and FSC1000 respectively were analysed using RFLP and clones were classified into 6, 6 and 5 groups for the respective stations. Two groups from FSC500 were represented by a single clone which did not return with valid sequences. Oceanospirillales were most abundant at all stations although the dominant genera were not shared between stations. *Alcanivorax*‐related clones covered 64% of all clones at FSC135, while *Marinomonas*‐ and *Oleispira*‐affiliated clones were dominant at FSC500 (40%) and FSC1000 (48·1%) respectively (Fig. 1). *Oleispira* was also abundant at FSC500 (27% of clones). The family Rhodobacteraceae constituted 11% of clones in FSC135 but were absent from the deep stations. A phylogenetic tree of sequenced clones and closest neighbour sequences is shown in Fig. 2.

### Crude oil degradation

**Figure 3:**
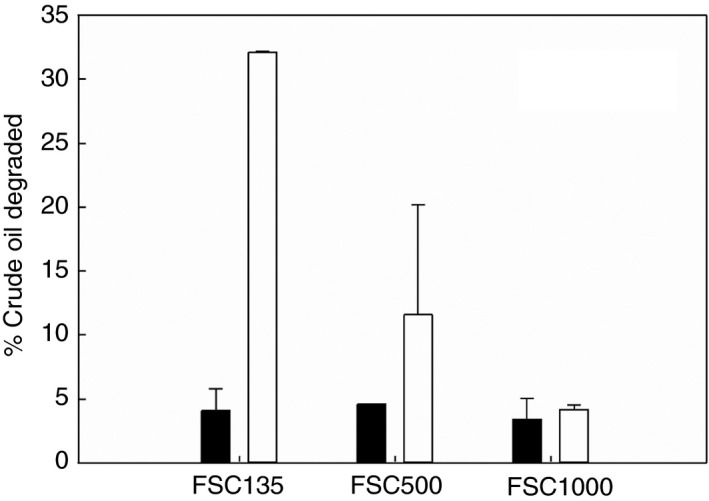
Percentage of crude oil degraded at each station after 3 weeks of incubation at ambient temperature (12, 5 and 0°C for FSC135, FSC500 and FSC1000 respectively). Treatments: control (black), inoculated (white).

Approximately 30% of added oil was degraded by the FSC135 consortium at the end of the 3‐week incubation period (Fig. 3). The difference between control and incubated flasks was statistically significant for FSC135 (*t*‐test, *P* = 0·001). The degradation of oil by FSC500 consortia ranged between 3–20% and was not significantly different from controls due to high variability between inoculated replicates. No significant crude oil degradation was measured in FSC1000 flasks.

## Discussion

Establishing environmental baselines and long‐term environmental monitoring are essential in order to assess natural environmental change and document anthropogenic perturbations. Lack of baseline data has been repeatedly identified as a major obstacle in assessing the ecological damage and recovery of the Gulf of Mexico following DWH, particularly where ultra‐deep drilling is now occurring (Joye *et al*. 2016). Such data are likewise generally unavailable for other parts of the world ocean where oil and gas exploration and drilling are ongoing including the FSC. This study is the first to provide baseline information on the diversity of crude oil‐degrading bacteria in deep‐water subarctic sediments. Both culture‐dependent and independent techniques were employed in order to capture as much diversity of hydrocarbon‐degrading bacteria as possible (Stefani *et al*. 2015). Clone libraries of enriched hydrocarbon‐degrading communities at all stations were dominated by Oceanospirillales and Alteromonadales; however, significant differences occurred at genus level particularly between the shallow and the deep, cold‐water stations. At FSC135, the community was dominated by *Alcanivorax* sp. which constituted 64% of clones after 3 weeks of incubation. It is reminded that the relative abundance of *Alcanivorax* in FSC135‐revived communities from the glycerol stocks used in this study was in the order of 3% (Potts *et al*. 2018). *Alcanivorax* was absent at deeper stations in agreement to observations from DWH where it was not readily detected in plume samples (Hazen *et al*. 2010; Mason *et al*. 2012; Rivers *et al*. 2013). More recently, comparisons of hydrocarbon‐degrading bacterial community dynamics between FSC135 and FSC1000, using diesel and model oil as growth substrates, further confirmed the absence of *Alcanivorax* from the deep station even at 20°C incubations (Potts *et al*. 2018). Available data suggest that *Alcanivorax* does not compete well with other taxa for deep‐water hydrocarbons and this may be attributed to its piezosensitivity (Kimes *et al*. 2013; Scoma *et al*. 2016a). The dominant Oceanospirillales, which was common between FSC500 and FSC1000, was *Oleispira. Oleispira* clones in this study were closely related to *O. antarctica*, a psychrophilic member of the obligate hydrocarbonoclastic bacteria group that is limited to cold waters in high latitudes and is capable of aliphatic hydrocarbon degradation (Yakimov *et al*. 2003). Although presence cannot be directly translated to activity, the relative abundance of *Oleispira* in revived communities from the same FSC1000 glycerol stocks used here was merely 0·002% after 30 h of incubation (Potts *et al*. 2018). This implies that *Oleispira* was actively growing in the enrichment cultures in this study and dominated the deep‐water hydrocarbon‐degrading communities after 3 weeks of incubation with crude oil. Despite being present and abundant at both deep stations, subzero temperatures at FSC1000 clearly favoured *Oleispira* as its relative abundance almost doubled compared to FSC500. Similarly, the genus *Oleispira* dominated the total and active bacterial population in oil‐contaminated sea ice during a field mesocosm experiment in Svalbard (Boccadoro *et al*. 2017) and showed intensive growth following contamination with crude oil of North Sea seawater (Krolicka *et al*. 2014) and deep water from the Gulf of Mexico (Techtmann *et al*. 2017). The ability of *Oleispira* to rapidly grow in the presence of oil and its ecological competitiveness in cold environments explain the ubiquity of *Oleispira*‐related Oceanospirillales in the world's oceans and render *Oleispira* a good candidate for developing biotechnological approaches for oil spill mitigation at near‐zero temperatures as those prevailing in the FSC and the poles (Kube *et al*. 2013). Nevertheless, this is a hypothesis that requires further testing as *Oleispira* does not always emerge as the dominant genus in cold environments following hydrocarbon contamination (Deppe *et al*. 2005; Garneau *et al*. 2016). It is noteworthy that our own experiments using a mixture of aliphatic and aromatic hydrocarbons instead of crude oil in FSC deep‐water sediment incubations did not reveal dominance of *Oleispira* at any stage of the degradation process, highlighting the importance of substrate type in shaping the response of the bacterial community structure to hydrocarbon contamination (Ferguson *et al*. 2017; Perez Calderon *et al*. 2018). The genus *Marinomonas* was detected in the FSC500 clone library only and was most abundant among Oceanospirillales at that station. Psychrotolerant members of *Marinomonas* have been previously identified in enriched oil‐degrading microbial communities from polar regions (Brakstad and Bonaunet 2006; Brakstad *et al*. 2008) but the individual role of this genus to oil degradation at low temperatures was not determined. *Marinomonas* isolated from shallow‐water temperate sediments have been shown to be capable of phenanthrene and chrysene degradation (Melcher *et al*. 2002). A *Marinomonas* strain isolated from FSC1000 in this study (97% similarity to *M. primoryensis*) was not capable of hydrocarbon degradation.

During DWH, Oceanospirillales were eventually supplanted by *Colwellia* (Alteromonadales) and *Cycloclasticus* (Thiotrichales) within a time frame of 2 weeks (Redmond and Valentine 2012; Yang *et al*. 2016b,b). *Colwellia* from the DWH deep‐water plume was capable of oil degradation at *in situ* temperature (Bælum *et al*. 2012) and single‐cell genomics revealed its capability for gaseous and aromatic hydrocarbon degradation, which was consistent with the observed succession pattern of the bacterial community (Mason *et al*. 2014). In this study, members of the genus *Colwellia* were present at all stations but were low in abundance, ranging from 2–14% of clones. Unlike *Colwellia* at 12°C (FSC135) and 5°C (FSC500), *Colwellia* clones from FSC1000 were closely related to psychrophilic strains of polar origin. The single *Colwellia* isolate in this study, which originated from FSC1000, tested negative for hydrocarbon degradation. Previous studies were also inconclusive about the role of *Colwellia* in oil degradation in the FSC; during a 50‐day incubation experiment of FSC sediments from 500 and 1000 m, *Colwellia* were abundant in both natural and oil‐contaminated sediments, which masked the possible role of this genus to hydrocarbon degradation (Ferguson *et al*. 2017). In a different study using undisturbed sediments from the deep FSC, *Colwellia* was detected in both control and hydrocarbon‐contaminated sediments, although, its relative abundance in the latter was twice that of controls (Perez Calderon *et al*. 2018). This is not surprising since *Colwellia* is a heterotrophic group that does not depend solely on external hydrocarbon supply for growth. Yang *et al*. (2016a) reported the presence of *Colwellia* in both oily and nonoily Gulf sediments collected 5–7 months after the DWH spill and also concluded that *Colwellia* could be autochthonous to deep surficial sediments and cannot be unambiguously linked to oil contamination. Other prevalent Alteromonadales in this study included *Pseudoalteromonas* particularly in clone libraries from FSC500 and FSC1000. *Pseudoalteromonas* was also one of the most abundant bacteria in both oil‐contaminated surface waters and the deep hydrocarbon plume during DWH (Dubinsky *et al*. 2013; Yang *et al*. 2016b). *Pseudoalteromonas* has been shown to respond rapidly—within 24 h—to oil contamination in 4°C mesocosm experiments with North Sea surface water (Krolicka *et al*. 2017). The significant role of *Pseudoalteromonas* in the early stages of oil biodegradation could be attributed to the EPS‐producing qualities of several members of this genus and their contribution to oil emulsification (Gutierrez *et al*. 2013). *Cycloclasticus* and *Thalassomonas*, two of the other genera identified as major oil‐degraders in the DWH deep‐water plume and in contaminated deep‐sea sediments in the Gulf of Mexico, were not present in clone libraries or isolated strains in this study (Bælum *et al*. 2012; Redmond and Valentine 2012; Dubinsky *et al*. 2013). During DWH, the family Rhodobacteraceae (Alphaproteobacteria) became enriched postspill and were associated with scavenging of the decaying bloom and consumption of organic residues in the plume (Dubinsky *et al*. 2013). It is thus not surprising that Rhodobacteraceae clones in this study were present in FSC135 only where the process of degradation was at a more advanced stage compared to the deeper stations.

Crude oil degradation by the oil‐degrading consortia after 3 weeks was significant only at FSC135 (12°C). No degradation was observed at FSC1000 at 0°C and high variability between replicates at FSC500 (5°C) did not return statistically significant differences between control and inoculated treatments. Nevertheless, most studies agree that oil biodegradation proceeds much slower at 0°C compared to 5°C and a longer lag period before the initiation of degradation is observed with decreasing temperature (Deppe *et al*. 2005; Brakstad and Bonaunet 2006; Ferguson *et al*. 2017). In this study, the apparent lack of measurable crude oil degradation at low temperatures after 20 days of incubation could be attributed to the combined effect of sampling occurring during the ‘adaptation’ period of the bacterial community and the low sensitivity of the gravimetric method. The significant presence of *Pseudoalteromonas* and *Oleispira* in FSC500 and FSC1000 consortia, both of which are known to respond rapidly to oil contamination at low temperatures, is an indication of the early successional stage of these communities (Krolicka *et al*. 2014, 2017; Techtmann *et al*. 2017).

Bacterial strains isolated in this study included several well‐known oil‐degrading taxa, such as the Gammaproteobacteria *Halomonas, Pseudomonas* and *Pseudoalteromonas* and the Actinobacterium *Rhodococcus* (Melcher *et al*. 2002; Dubinsky *et al*. 2013; Gutierrez *et al*. 2013). *Rhodococcus* was the only common genus among isolates from all three stations and returned a strong signal in hydrocarbon degradation testing. Indeed, members of *Rhodococcus* are known as efficient alkane degraders (Sorkhoh *et al*. 1990; Yakimov *et al*. 1999; Sharma and Pant 2000). Apart from a direct role in hydrocarbon degradation, *Rhodococcus* produce extracellular polysaccharides that have been shown to facilitate the biodegradation of the aromatic fraction of crude oil by other bacteria (Iwabuchi *et al*. 2002). In addition to bacterial taxa commonly associated with oil contamination, an extensive list of less known bacterial oil‐degraders was produced in this study, several of which are reported in that capacity for the first time. The Alphaproteobacteria *Sulfitobacter* and *Ahrensia,* the Flavobacterium *Belliella* sp. and an Actinobacterium of the Kineosporiaceae family (95% similarity to *Pseudokineococcus lusitanus*) from FSC135 tested positive for hydrocarbon degradation. To our knowledge, none of these genera are known as active hydrocarbon degraders *per se*;* Sulfitobacter* has been previously reported as one of the most abundant bacteria in biodegradation experiments with oil‐contaminated North Sea seawater (Krolicka *et al*. 2017) and AlkB genes related to *Ahrensia* have been found in marine metagenomes (Nie *et al*. 2014). *Chryseobacterium* sp. was unique to FSC500 and showed strong ability for hydrocarbon degradation. Similarly, no representatives of this genus have been described previously as capable of hydrocarbon degradation.

*Pseudoalteromonas, Shewanella* and *Glaciecola* isolates were unique to deep stations. These genera include several psychrophilic and psychrotolerant representatives isolated from polar regions with several *Shewanella* also being piezotolerant i.e. growing in high‐pressure environments (DeLong *et al*. 1997; Bowman *et al*. 1998; Kato and Nogi 2001; Van Trappen *et al*. 2004; Zhang *et al*. 2006). Nevertheless, both *Shewanella* and *Glaciecola* strains tested negative for oil degradation in this study. Similarly, a *Shewanella* isolate from a crude oil‐enriched arctic microbial consortium was unable to significantly degrade crude oil in monoculture (Deppe *et al*. 2005) and *Shewanella* from oil‐contaminated Arctic sea‐ice samples showed only weak ability to degrade hexadecane but not toluene (Gerdes *et al*. 2005). *Glaciecola* strains isolated from oil‐contaminated sites along the Norwegian coast have been identified as biosurfactant producers, thereby contributing to the emulsification process (Dang *et al*. 2016).

Overall, this study provided a first account of key crude oil‐degrading bacteria in sediments of the FSC, an area of ultra‐deep drilling on the UKCS. The list of hydrocarbon‐degrading bacterial isolates generated in this study included several strains that were reported for the first time in that capacity. The genus *Oleispira* emerged as a major player in the early stages of crude oil degradation in deep‐sea sediments particularly at subzero temperatures. This finding is offering a direction of future research into biomonitoring tools for the detection of low levels of crude oil contamination in cold‐water ecosystems.

## Conflict of Interest

No conflict of interest declared.

## Supporting information


**Table S1.** Summary of enrichment culture treatments and volume of PAH stock solution and crude oil added in each transfer.

## References

[jam14030-bib-0001] Austin, J.A. , Cannon, S.J.C. and Ellis, D. (2014) Hydrocarbon exploration and exploitation West of Shetlands. Geological Society, London, Special Publications 397, 1–10.

[jam14030-bib-0002] Bælum, J. , Borglin, S. , Chakraborty, R. , Fortney, J.L. , Lamendella, R. , Mason, O.U. , Auer, M. , Zemla, M. *et al* (2012) Deep‐sea bacteria enriched by oil and dispersant from the Deepwater Horizon spill. Environ Microbiol 14, 2405–2416. 22616650 10.1111/j.1462-2920.2012.02780.x

[jam14030-bib-0003] Berx, B. (2012) The hydrography and circulation of the Faroe‐Shetland Channel. Ocean Challenge 19, 15–19.

[jam14030-bib-0004] Boccadoro, K. , Krolicka, A. , Chever, F. , Ramanand, S. , Austerheim, E. and Le Floch, S. (2017) Biodegradation of crude oil and impact on arctic microbial populations in sea ice and seawater following in situ oil spill treatments in Svalbard. In: ISMOS 6 Abstract Book, p. 34.

[jam14030-bib-0005] Bowman, J.P. , MCcammon, S.A. , Brown, J.L. and Mcmeekin, T.A. (1998) *Glaciecola punicea* gen. nov., sp. nov. and *Glaciecola pallidula* gen. nov., sp. nov. : psychrophilic bacteria from Antarctic sea‐ice habitats. Int J Syst Bacteriol 48, 1213–1222. 10.1099/00207713-47-3-670 9226898

[jam14030-bib-0006] Brakstad, O.G. and Bonaunet, K. (2006) Biodegradation of petroleum hydrocarbons in seawater at low temperatures (0–5 & #xB0;C) and bacterial communities associated with degradation. Biodegradation 17, 71–82. 16453173 10.1007/s10532-005-3342-8

[jam14030-bib-0007] Brakstad, O.G. , Nonstad, I. , Faksness, L.‐G. and Brandvik, P.J. (2008) Responses of microbial communities in Arctic sea ice after contamination by crude petroleum oil. Microb Ecol 55, 540–552. 17805918 10.1007/s00248-007-9299-x

[jam14030-bib-0008] Brooks, G.R. , Larson, R.A. , Schwing, P.T. , Romero, I. , Moore, C. , Reichart, G.‐J. , Jilbert, T. , Chanton, J.P. *et al* (2015) Sedimentation pulse in the NE Gulf of Mexico following the 2010 DWH Blowout. PLoS ONE 10, e0132341. 26172639 10.1371/journal.pone.0132341 PMC4501746

[jam14030-bib-0009] Campo, P. , Venosa, A.D. and Suidan, M.T. (2013) Biodegradability of Corexit 9500 and dispersed South Louisiana Crude Oil at 5 and 25°C. Environ Sci Technol 47, 1960–1967. 23363064 10.1021/es303881h

[jam14030-bib-0010] Chanton, J. , Zhao, T. , Rosenheim, B.E. , Joye, S. , Bosman, S. , Brunner, C. , Yeager, K.M. , Diercks, A.R. *et al* (2014) Using natural abundance radiocarbon to trace the flux of petrocarbon to the seafloor following the Deepwater Horizon oil spill. Environ Sci Technol 49, 847–854. 10.1021/es5046524 25494527

[jam14030-bib-0011] Cole, J.R. , Wang, Q. , Fish, J.A. , Chai, B. , McGarrell, D.M. , Sun, Y. , Brown, C.T. , Porras‐Alfaro, A. *et al* (2014) Ribosomal database project: data and tools for high throughput rRNA analysis. Nucleic Acids Res 42, D633–D642. 24288368 10.1093/nar/gkt1244 PMC3965039

[jam14030-bib-0012] Cordes, E.E. , Jones, D.O.B. , Schlacher, T.A. , Amon, D.J. , Bernardino, A.F. , Brooke, S. , Carney, R. , DeLeo, D.M. *et al* (2016) Environmental mpacts of the deep‐water oil and gas industry: a review to guide management strategies. Front Environ Sci 4, 1–54.

[jam14030-bib-0013] Daly, K.L. , Passow, U. , Chanton, J. and Hollander, D. (2016) Assessing the impacts of oil‐associated marine snow formation and sedimentation during and after the Deepwater Horizon oil spill. Anthropocene 13, 18–33.

[jam14030-bib-0014] Dang, N.P. , Landfald, B. and Willassen, N.P. (2016) Biological surface‐active compounds from marine bacteria. Environ Technol 37, 1151–1158. 26506920 10.1080/09593330.2015.1103784

[jam14030-bib-0015] DeLong, E.F. , Franks, D.G. and Yayanos, A.A. (1997) Evolutionary relationships of cultivated psychrophilic and barophilic deep‐sea bacteria. Appl Environ Microbiol 63, 2105–2108. 16535621 10.1128/aem.63.5.2105-2108.1997 PMC1389176

[jam14030-bib-0016] Deppe, U. , Richnow, H.‐H. , Michaelis, W. and Antranikian, G. (2005) Degradation of crude oil by an arctic microbial consortium. Extremophiles 9, 461–470. 15999222 10.1007/s00792-005-0463-2

[jam14030-bib-0017] Dubinsky, E.A. , Conrad, M.E. , Chakraborty, R. , Bill, M. , Borglin, S.E. , Hollibaugh, J.T. , Mason, O.U. , M Piceno, Y. *et al* (2013) Succession of hydrocarbon‐degrading bacteria in the aftermath of the deepwater horizon oil spill in the gulf of Mexico. Environ Sci Technol 47, 10860–10867. 23937111 10.1021/es401676y

[jam14030-bib-0018] Dyksterhouse, S.E. , Gray, J.P. , Herwig, R.P. , Lara, J.C. and Staley, J.T. (1995) *Cycloclasticus pugetii* gen. nov., sp. nov., an aromatic hydrocarbon‐degrading bacterium from marine sediments. Int J Syst Bacteriol 45, 116–123. 7857792 10.1099/00207713-45-1-116

[jam14030-bib-0019] Ferguson, R.M.W. , Gontikaki, E. , Anderson, J.A. and Witte, U. (2017) The variable influence of dispersant on degradation of oil hydrocarbons in subarctic deep‐sea sediments at low temperatures (0–5 & #xB0;C). Sci Rep 7, 2253. 28533547 10.1038/s41598-017-02475-9 PMC5440406

[jam14030-bib-0999] Garneau, M‐E. , Michel, C. , Meisterhans, G. , Fortin, N. , King, T.L. , Greer, C.W. and Lee, K. (2016) Hydrocarbon biodegradation by Arctic ea‐ice and sub‐ice microbial communities during microcosm experiments, Northwest Passage (Nunavut, Canada). FEMS Microbiol Ecol 92, fiw130. 27387912 10.1093/femsec/fiw130

[jam14030-bib-0020] Gerdes, B. , Brinkmeyer, R. , Dieckmann, G. and Helmke, E. (2005) Influence of crude oil on changes of bacterial communities in Arctic sea‐ice. FEMS Microbiol Ecol 53, 129–139. 16329935 10.1016/j.femsec.2004.11.010

[jam14030-bib-0021] Gutierrez, T. , Singleton, D.R. , Berry, D. , Yang, T. , Aitken, M.D. and Teske, A. (2013) Hydrocarbon‐degrading bacteria enriched by the Deepwater Horizon oil spill identified by cultivation and DNA‐SIP. The ISME J 7, 2091–2104. 23788333 10.1038/ismej.2013.98 PMC3806270

[jam14030-bib-0022] Hazen, T.C. , Dubinsky, E.A. , DeSantis, T.Z. , Andersen, G.L. , Piceno, Y.M. , Singh, N. , Jansson, J.K. , Probst, A. *et al* (2010) Deep‐sea oil plume enriches indigenous oil‐degrading bacteria. Science 330, 204–208. 20736401 10.1126/science.1195979

[jam14030-bib-0023] Iwabuchi, N. , Sunairi, M. , Urai, M. , Itoh, C. , Anzai, H. , Nakajima, M. and Harayama, S. (2002) Extracellular polysaccharides of *Rhodococcus rhodochrous* S‐2 stimulate the degradation of aromatic components in crude oil by indigenous marine bacteria. Appl Environ Microbiol 68, 2337–2343. 11976106 10.1128/AEM.68.5.2337-2343.2002 PMC127525

[jam14030-bib-0024] Jernelöv, A. (2010) How to defend against future oil spills. Nature 466, 182–183. 20613822 10.1038/466182a

[jam14030-bib-0025] Joye, S.B. (2015) Deepwater Horizon, 5 years on. Science 349, 592–593. 26250675 10.1126/science.aab4133

[jam14030-bib-0026] Joye, S.B. , Teske, A.P. and Kostka, J.E. (2014) Microbial dynamics following the macondo oil well blowout across gulf of Mexico environments. Bioscience 64, 766–777.

[jam14030-bib-0027] Joye, S.B. , Kleindienst, S. , Gilbert, J.A. , Handley, K.M. , Weisenhorn, P. , Overholt, W.A. and Kostka, J.E. (2016) Responses of microbial communities to hydrocarbon exposures. Oceanography 29, 136–149.

[jam14030-bib-0028] Kato, C. and Nogi, Y. (2001) Correlation between phylogenetic structure and function: Examples from deep‐sea *Shewanella* . FEMS Microbiol Ecol 35, 223–230. 11311432 10.1111/j.1574-6941.2001.tb00807.x

[jam14030-bib-0029] Kimes, N.E. , Callaghan, A.V. , Aktas, D.F. , Smith, W.L. , Sunner, J. , Golding, B. , Drozdowska, M. , Hazen, T.C. *et al* (2013) Metagenomic analysis and metabolite profiling of deep‐sea sediments from the Gulf of Mexico following the Deepwater Horizon oil spill. Front Microbiol 4 Article 50, 1–17. 23508965 10.3389/fmicb.2013.00050 PMC3598227

[jam14030-bib-0030] Kimura, M. (1980) A simple method for estimating evolutionary rate of base substitutions through comparative studies of nucleotide sequences. J Mol Evol 16, 111–120. 7463489 10.1007/BF01731581

[jam14030-bib-0031] Krolicka, A. , Boccadoro, C. , Mealand, M. , Preston, C. , Birch, J.. , Scholin, C. and Baussant, T. (2014) Detection of oil leaks by quantifying hydrocarbonoclastic bacteria in cold marine environments using the environmental sample processor In 37th AMOP Technical Seminar on Environmental Contamination and Response, pp. 791–807.

[jam14030-bib-0032] Krolicka, A. , Boccadoro, C. , Nilsen, M.M. and Baussant, T. (2017) Capturing early changes in the marine bacterial community as a result of crude oil pollution in a mesocosm experiment. Microbes Environ 32, 358–366. 29187706 10.1264/jsme2.ME17082 PMC5745021

[jam14030-bib-0033] Kube, M. , Chernikova, T.N. , Al‐Ramahi, Y. , Beloqui, A. , Lopez‐Cortez, N. , Guazzaroni, M.‐E. , Heipieper, H.J. , Klages, S. *et al* (2013) Genome sequence and functional genomic analysis of the oil‐degrading bacterium *Oleispira antarctica* . Nature Commun 4, 2156. 23877221 10.1038/ncomms3156 PMC3759055

[jam14030-bib-0034] Kumar, S. , Stecher, G. and Tamura, K. (2016) MEGA7: molecular evolutionary genetics analysis version 7.0 for bigger datasets. Mol Biol Evol 33, 1870–1874. 27004904 10.1093/molbev/msw054 PMC8210823

[jam14030-bib-0035] Lane, D.J. (1991) *16S/23S rRNA sequencing* In Nucleic Acid Techniques in Bacterial Systematics ed. Stackebrandt E. and Goodfellow M. pp. 115–175. New York, NY: John Wiley and Sons.

[jam14030-bib-0036] Larkin, K.E. , Donaldson, K. , McDonough, N. and Rogers, A. (2015) Delving Deeper: How Can We Achieve Sustainable Management of Our Deep Sea Through Integrated Research. EMB Policy. Ostend, Belgium: European Marine Board.

[jam14030-bib-0037] Leigh, M.B. , Taylor, L. and Neufeld, J.D. (2010) *Clone libraries of ribosomal RNA gene sequences for characterization of bacterial and fungal communities* In Handbook of Hydrocarbon and Lipid Microbiology ed. Timmis K., McGenity T., van der Meer J. and de Lorenzo V. pp. 3969–3993. Berlin Heidelberg: Springer‐Verlag.

[jam14030-bib-0038] Mason, O.U. , Hazen, T.C. , Borglin, S. , Chain, P.S.G. , Dubinsky, E.A. , Fortney, J.L. , Han, J. , Holman, H.‐Y.N. *et al* (2012) Metagenome, metatranscriptome and single‐cell sequencing reveal microbial response to Deepwater Horizon oil spill. The ISME J 6, 1715–1727. 22717885 10.1038/ismej.2012.59 PMC3498917

[jam14030-bib-0039] Mason, O.U. , Han, J. , Woyke, T. and Jansson, J.K. (2014) Single‐cell genomics reveals features of a *Colwellia* species that was dominant during the Deepwater Horizon oil spill. Front Microbiol 5, 332. 25071745 10.3389/fmicb.2014.00332 PMC4085564

[jam14030-bib-0040] Melcher, R.J. , Apitz, S.E. and Hemmingsen, B.B. (2002) Impact of irradiation and polycyclic aromatic hydrocarbon spiking on microbial populations in marine sediment for future aging and biodegradability studies. Appl Environ Microbiol 68, 2858–2868. 12039743 10.1128/AEM.68.6.2858-2868.2002 PMC123915

[jam14030-bib-0041] Montagna, P.A. , Baguley, J.G. , Cooksey, C. , Hartwell, I. , Hyde, L.J. , Hyland, J.L. , Kalke, R.D. , Kracker, L.M. *et al* (2013) Deep‐sea benthic footprint of the Deepwater Horizon blowout. PLoS ONE 8, e70540. 23950956 10.1371/journal.pone.0070540 PMC3737147

[jam14030-bib-0042] Muehlenbachs, L. , Cohen, M.A. and Gerarden, T. (2013) The impact of water depth on safety and environmental performance in offshore oil and gas production. Energy Pol 55, 699–705.

[jam14030-bib-0043] Nie, Y. , Chi, C.‐Q. , Fang, H. , Liang, J.‐L. , Lu, S.‐L. , Lai, G.‐L. , Tang, Y.‐Q. and Wu, X.‐L. (2014) Diverse alkane hydroxylase genes in microorganisms and environments. Sci Rep 4, 1552–1560. 10.1038/srep04968 PMC4021335 24829093

[jam14030-bib-0044] Passow, U. and Hetland, R.D. (2016) What happened to all of the oil? Oceanography 29, 88–95.

[jam14030-bib-0045] Passow, U. , Ziervogel, K. , Asper, V. and Diercks, A. (2012) Marine snow formation in the aftermath of the Deepwater Horizon oil spill in the Gulf of Mexico. Environ Res Lett 7, 35301.

[jam14030-bib-0046] Perez Calderon, L.J. , Potts, L.D. , Gontikaki, E. , Gubry‐Rangin, C. , Cornulier, T. , Gallego, A. , Anderson, J.A. and Witte, U. (2018) Bacterial community response in deep Faroe‐Shetland Channel sediments following hydrocarbon entrainment with and without dispersant addition. Front Mar Sci 5, 159.

[jam14030-bib-0444] Potts, L.D. , Perez Calderon, L.J. , Gontikaki, E. , Keith, L. , Gubry‐Rangin, C. , Anderson, J.A. and Witte, U.A. (2018) Effect of spatial origin and hydrocarbon composition on bacterial consortia community structure and hydrocarbon biodegradation rates. FEMS Microbiol Ecol, fiy127 (accepted) 10.1093/femsec/fiy127 PMC6166136 29982504

[jam14030-bib-0047] Prakash, O. , Nimonkar, Y. and Shouche, Y.S. (2013) Practice and prospects of microbial preservation. FEMS Microbiol Lett 339, 1–9. 23083094 10.1111/1574-6968.12034

[jam14030-bib-0048] Pruesse, E. , Quast, C. , Knittel, K. , Fuchs, B.M. , Ludwig, W. , Peplies, J. and Glöckner, F.O. (2007) SILVA: a comprehensive online resource for quality checked and aligned ribosomal RNA sequence data compatible with ARB. Nucleic Acids Res 35, 7188–7196. 17947321 10.1093/nar/gkm864 PMC2175337

[jam14030-bib-0049] Redmond, M. and Valentine, D. (2012) Natural gas and temperature structured a microbial community response to the Deepwater Horizon oil spill. Proc Natl Acad Sci USA 109, 20292–20297. 21969552 10.1073/pnas.1108756108 PMC3528494

[jam14030-bib-0050] Rivers, A.R. , Sharma, S. , Tringe, S.G. , Martin, J. , Joye, S.B. and Moran, M.A. (2013) Transcriptional response of bathypelagic marine bacterioplankton to the Deepwater Horizon oil spill. The ISME J 7, 2315–2329. 23902988 10.1038/ismej.2013.129 PMC3834857

[jam14030-bib-0051] Romero, I.C. , Schwing, P.T. , Brooks, G.R. , Larson, R.A. , Hastings, D.W. , Ellis, G. , Goddard, E.A. and Hollander, D.J. (2015) Hydrocarbons in deep‐sea sediments following the 2010 Deepwater Horizon blowout in the northeast Gulf of Mexico. PLoS ONE 10, e0128371. 26020923 10.1371/journal.pone.0128371 PMC4447447

[jam14030-bib-0052] Schwing, P.T. , Romero, I.C. , Brooks, G.R. , Hastings, D.W. , Larson, R.A. and Hollander, D.J. (2015) A decline in benthic foraminifera following the deepwater horizon event in the northeastern Gulf of Mexico. PLoS ONE 10, e0120565. 25785988 10.1371/journal.pone.0120565 PMC4364910

[jam14030-bib-0054] Scoma, A. , Yakimov, M.M. and Boon, N. (2016a) Challenging oil bioremediation at deep‐sea hydrostatic pressure. Front Microbiol 7, 1203. 27536290 10.3389/fmicb.2016.01203 PMC4971052

[jam14030-bib-0053] Scoma, A. , Barbato, M. , Hernandez‐Sanabria, E. , Mapelli, F. , Daffonchio, D. , Borin, S. and Boon, N. (2016b) Microbial oil‐degradation under mild hydrostatic pressure (10 MPa): which pathways are impacted in piezosensitive hydrocarbonoclastic bacteria? Sci Rep 6, 23526. 27020120 10.1038/srep23526 PMC4810429

[jam14030-bib-0055] Sharma, S.L. and Pant, A. (2000) Biodegradation and conversion of alkanes and crude oil by a marine *Rhodococcus* sp. Biodegradation 11, 289–294. 11487058 10.1023/a:1011185806974

[jam14030-bib-0056] Sorkhoh, N.A. , Ghannoum, M.A. , Ibrahim, A.S. , Stretton, R.J. and Radwan, S.S. (1990) Crude oil and hydrocarbon‐degrading strains of *Rhodococcus rhodochrous* isolated from soil and marine environments in Kuwait. Environ Poll 65, 1–17. 10.1016/0269-7491(90)90162-6 15092275

[jam14030-bib-0057] Stefani, F.O.P. , Bell, T.H. , Marchand, C. , de la Providencia, I.E. , El Yassimi, A. , St‐Arnaud, M. and Hijri, M. (2015) Culture‐dependent and ‐independent methods capture different microbial community fractions in hydrocarbon‐contaminated soils. PLoS ONE 10, e0128272. 26053848 10.1371/journal.pone.0128272 PMC4460130

[jam14030-bib-0058] Stucky, B.J. (2012) Seqtrace: a graphical tool for rapidly processing DNA sequencing chromatograms. J Biomol Tech 23, 90–93. 22942788 10.7171/jbt.12-2303-004 PMC3413935

[jam14030-bib-0059] Techtmann, S.M. , Zhuang, M. , Campo, P. , Holder, E. , Elk, M. , Hazen, T.C. , Conmy, R. and Santo Domingo, J.W. (2017) Corexit 9500 enhances oil biodegradation and changes active bacterial community structure of oil‐enriched microcosms. Appl Environ Microbiol 83, e03462‐16. 28283527 10.1128/AEM.03462-16 PMC5411496

[jam14030-bib-0060] Valentine, D.L. , Fisher, G.B. , Bagby, S.C. , Nelson, R.K. , Reddy, C.M. , Sylva, S.P. and Woo, M.A. (2014) Fallout plume of submerged oil from Deepwater Horizon. Proc Natl Acad Sci USA 111, 15906–15911. 25349409 10.1073/pnas.1414873111 PMC4234598

[jam14030-bib-0061] Van Trappen, S. , Tan, T.L. , Yang, J. , Mergaert, J. and Swings, J. (2004) *Glaciecola polaris* sp. nov., a novel budding and prosthecate bacterium from the Arctic Ocean, and emended description of the genus *Glaciecola* . Int J Syst Evol Microbiol 54, 1765–1771. 15388742 10.1099/ijs.0.63123-0

[jam14030-bib-0062] Vergin, K.L. , Urbach, E. , Stein, J.L. , DeLong, E.F. , Lanoil, B.D. and Giovannoni, S.J. (1998) Screening of a fosmid library of marine environmental genomic DNA fragments reveals four clones related to members of the order Planctomycetales. Appl Environ Microbiol 64, 3075–3078. 9687477 10.1128/aem.64.8.3075-3078.1998 PMC106819

[jam14030-bib-0063] Vergin, K.L. , Rappé, M.S. and Giovannoni, S.J. (2001) Streamlined method to analyze 16S rRNA gene clone libraries. Biotechniques 30, 938–944. 11355353 10.2144/01305bm03

[jam14030-bib-0064] Wrenn, B.A. and Venosa, A.D. (1996) Selective enumeration of aromatic and aliphatic hydrocarbon degrading bacteria by a most‐probable‐number procedure. Can J Microbiol 42, 252–258. 8868232 10.1139/m96-037

[jam14030-bib-0065] Yakimov, M.M. , Giuliano, L. , Bruni, V. , Scarfi, S. and Golyshin, P.N. (1999) Characterization of Antarctic hydrocarbon‐degrading bacteria capable of producing bioemulsifiers. New Microbiol 22, 249–256. 10423744

[jam14030-bib-0066] Yakimov, M.M. , Giuliano, L. , Gentile, G. , Crisafi, E. , Chernikova, T.N. , Abraham, W.R. , Lünsdorf, H. , Timmis, K.N. *et al* (2003) *Oleispira antarctica* gen. nov., sp. nov., a novel hydrocarbonoclastic marine bacterium isolated from Antarctic coastal sea water. Int J Syst Evol Microbiol 53, 779–785. 12807200 10.1099/ijs.0.02366-0

[jam14030-bib-0068] Yan, B. , Passow, U. , Chanton, J.P. , Nöthig, E.‐M. , Asper, V. , Sweet, J. , Pitiranggon, M. , Diercks, A. et al. (2016) Sustained deposition of contaminants from the Deepwater Horizon spill. Proc Natl Acad Sci USA 113, E3332–E3340. 27247393 10.1073/pnas.1513156113 PMC4914201

[jam14030-bib-0070] Yang, T. , Speare, K. , McKay, L. , MacGregor, B.J. , Joye, S.B. and Teske, A. (2016a) Distinct bacterial communities in surficial seafloor sediments following the 2010 Deepwater Horizon blowout. Front Microbiol 7, 1384. 27679609 10.3389/fmicb.2016.01384 PMC5020131

[jam14030-bib-0069] Yang, T. , Nigro, L.M. , Gutierrez, T. , D׳Ambrosio, L. , Joye, S.B. , Highsmith, R. and Teske, A. (2016b) Pulsed blooms and persistent oil‐degrading bacterial populations in the water column during and after the Deepwater Horizon blowout. Deep Sea Res II 129, 282–291.

[jam14030-bib-0071] Yu, C. , Reddy, A.P. , Simmons, C.W. , Simmons, B.A. , Singer, S.W. and Vander Gheynst, J.S. (2015) Preservation of microbial communities enriched on lignocellulose under thermophilic and high‐solid conditions. Biotechnol Biofuels 8, 1–13. 26633993 10.1186/s13068-015-0392-y PMC4667496

[jam14030-bib-0072] Zhang, D.C. , Yu, Y. , Chen, B. , Wang, H.X. , Liu, H.C. , Dong, X.Z. and Zhou, P.J. (2006) *Glaciecola psychrophila* sp. nov., a novel psychrophilic bacterium isolated from the Arctic. Int J Syst Evol Microbiol 56, 2867–2869. 17158989 10.1099/ijs.0.64575-0

[jam14030-bib-0073] Ziervogel, K. , Joye, S.B. and Arnosti, C. (2016) Microbial enzymatic activity and secondary production in sediments affected by the sedimentation pulse following the Deepwater Horizon oil spill. Deep Sea Res II 129, 241–248.

