# Supplementary material for: Hydrocarbon‐degrading bacteria in deep‐water subarctic sediments (Faroe‐Shetland Channel)
Source: J Appl Microbiol. 2018 Jul 24;125(4):1040–53. doi: 10.1111/jam.14030 (PMC6849767; doi:10.1111/jam.14030)
Supplement: Supplementary file 1 — Table S1. Summary of enrichment culture treatments and volume of PAH stock solution and crude oil added in each transfer. [file JAM-125-1040-s001.docx]

Supporting information

**Table S1.** Summary of enrichment culture treatments and volume of PAH stock solution and crude oil added in each transfer.

| **Treatment flasks** | **Volume (ul) of stock solution (10 mg ml^-1^)** | | |
| --- | --- | --- | --- |
|  | INITIAL ENRICHMENT | 1^ST^ TRANSFER | 2^ND^ TRANSFER |
| NAP | 90 | 135 | 180 |
| PHE | 90 | 135 | 180 |
| PYR | 90 | 135 | 180 |
| PAH mix* | 20 | 30 | 40 |
| Crude oil | 300 | 300 | 300 |

*Values indicate amount of individual PAHs in the PAH mix. The PAH mix consisted of 5 compounds: naphthalene, phenanthrene, pyrene, anthracene, fluorene.
